# A Low-Tech Analytical Method for Diethylcarbamazine Citrate in Medicated Salt

**DOI:** 10.1371/journal.pntd.0001005

**Published:** 2011-02-08

**Authors:** Abigail Weaver, Patrick Brown, Shannon Huey, Marco Magallon, E. Brennan Bollman, Dominique Mares, Thomas G. Streit, Marya Lieberman

**Affiliations:** 1 Department of Chemistry and Biochemistry, University of Notre Dame, Notre Dame, Indiana, United States of America; 2 Brigham Young University, Provo, Utah, United States of America; 3 Group SPES, Port-au-Prince, Haiti; 4 Department of Biology, University of Notre Dame, Notre Dame, Indiana, United States of America; McGill University, Canada

## Abstract

The World Health Organization has called for an effort to eliminate Lymphatic Filariasis (LF) around the world. In regions where the disease is endemic, local production and distribution of medicated salt dosed with diethylcarbamazine (DEC) has been an effective method for eradicating LF. A partner of the Notre Dame Haiti program, Group SPES in Port-au-Prince, Haiti, produces a medicated salt called Bon Sel. Coarse salt is pre-washed and sprayed with a solution of DEC citrate and potassium iodate. Iodine levels are routinely monitored on site by a titrimetric method. However, the factory had no method for monitoring DEC. Critical analytical issues include 1) determining whether the amount of DEC in each lot of Bon Sel is within safe and therapeutically useful limits, 2) monitoring variability within and between production runs, and 3) determining the effect of a common local practice (washing salt before use) on the availability of DEC. This paper describes a novel titrimetric method for analysis of DEC citrate in medicated salt. The analysis needs no electrical power and requires only a balance, volumetric glassware, and burets that most salt production programs have on hand for monitoring iodine levels. The staff of the factory used this analysis method on site to detect underloading of DEC on the salt by their sprayer and to test a process change that fixed the problem.

## Author Summary

As researchers develop more sophisticated technologies, parts of the world are left behind. The front lines of fighting many diseases lie in regions where expensive technology is not feasible. As part of the effort to eradicate lymphatic filariasis in Haiti, our group's goal was to design an assay that would allow a chemist, with basic equipment, to quantify the levels of diethylcarbamazine citrate on medicated salt. With access to university research facilities, we were able to devise and test a back-titration procedure that can measure the medication levels with sufficient accuracy and precision. Our method capitalized on the fact that the medication is acidic. This characteristic allows us to combine an unknown, medicated salt sample with a known quantity of base and then back-titrate with acid to determine diethylcarbamazine citrate concentration based on the neutralization point. Developing this protocol has put the power of quality control into the hands of the Haitian factory producing the medicated salt. With the ability to better monitor dosing levels, we have increased the effectiveness of this program in Haiti. Using modern research facilities to produce effective, low-tech methods could be a useful approach for tackling many worldwide medical and environmental issues.

## Introduction

The World Health Organization has called for an effort to eliminate Lymphatic Filariasis (LF) around the world. [1] A nematode worm (*Wuchereria bancrofti*) is the cause of 90% of lymphatic filariasis cases globally. Mosquito bites transmit larval nematodes (microfilariae) present in the blood stream of infected persons, and although the adult nematodes are resistant to medical treatment, human transmission in endemic regions can be stopped by administering drugs, such as diethylcarbamazine (DEC), that kill the microfilariae. DEC has had a long history of safe use in mass drug administration (MDA) LF eradication programs, [2]–[4] and so far, *W. bancrofti* do not appear to have developed resistance to DEC. [5]–[6] A course of treatment of 6 mg/kg per day of DEC citrate for 12 days (daily dose around 300 mg) can significantly reduce the microfilariae count in an infected person. However, in regions where the disease is endemic, yearly drug administration to infected individuals must be continued over the adult worm lifetime of 4–6 years to eradicate the disease. As an alternative to pill-based MDA, DEC can be administered to local populations in the form of medicated cooking salt, with DEC citrate present at 0.2–0.4% w/w, which corresponds to a daily dose of 20–40 mg DEC citrate. Local production and distribution of medicated salt fortified with DEC has proved to be a particularly effective method [7]–[8] for eradicating LF from endemic regions [9]–[10].

A partner of the Notre Dame Haiti program, Group SPES in Port-au-Prince, Haiti, produces a double-supplemented salt called “Bon Sel”. [11] Coarse salt is pre-washed and sprayed with a solution of DEC citrate and potassium iodate. Iodine levels are routinely monitored on site by a titrimetric method. However, as of 2010, the factory had no analytical process for monitoring DEC levels. Critical analytical issues include 1) determining whether the amount of DEC citrate in each lot of Bon Sel is within safe and therapeutically useful limits, 2) monitoring variability within and between production runs, and 3) determining the effect of a common local practice (washing salt before use) on the availability of DEC.

The “gold standard” assay for DEC citrate uses high-performance liquid chromatography (HPLC). [12] Sending samples out for analysis would impose unwanted costs and prevent real time analysis of production runs, yet it was impossible to implement this process at the factory in Haiti, which has no access to an HPLC or to the supplies and expertise necessary to maintain one. Color tests and spectrophotometry have been used for monitoring DEC-medicated salt production, [13]–[15] although usually for qualitative monitoring. [16] The facility in Haiti wanted quantitative information but did not have a spectrometer. The goal of our group was to develop a back titration assay for DEC citrate in medicated salt requiring only a balance, volumetric glassware, and burets, equipment that most iodized salt production programs have on hand for monitoring iodine levels, and compare this method against the benchmark HPLC method.

## Materials and Methods

### Materials

Samples of untreated NaCl and pharmaceutical grade DEC citrate (EPICO) were obtained from the Bon Sel plant in Haiti; pure DEC citrate for HPLC standardization was obtained from Sigma-Aldrich. The untreated NaCl was a coarse grade produced by evaporation of seawater and had visible contaminants (dirt, sand, plant matter).

0.0040 M HCl was prepared by sequential volumetric dilution of concentrated HCl, and stored in a plastic bottle. Dilute NaOH solutions are unstable due to reaction with atmospheric CO_2_. A 0.200 M NaOH stock solution should be prepared (it is stable for at least 4 weeks) and diluted each day to give the working 0.0100 M NaOH solution. Phenolphthalein indicator solution was prepared by dissolving 0.5 g of phenolphthalein (Aldrich) in 500 mL of a 50% ethanol:water solution.

Standards: DEC citrate standards (0.05%, 0.125%, 0.25%, and 0.50% w/w of salt) are prepared in the same matrix as the medicated salt samples. The final solutions are 10% w/v in salt, thus, to prepare the 0.50% standard, 10 g NaCl and 0.0500 g DEC citrate are mixed with DI water to give a final volume of 100 ml.

Samples: 5.00 g of medicated salt is dissolved in deionized or distilled water to a final volume of 50.00 ml with vigorous shaking (or 10 g/100 ml final volume). A small amount of insoluble residue is usually present in these samples.

#### NMR characterization of DEC citrate

DEC citrate in D_2_O (∼16 mg/ml, 400 MHz, shifts in ppm vs. TMS): 3.66 (d, 2H, J = 13.2 Hz, piperazine ring proton); 3.42 (d, 2H, J = 11.2 Hz, piperazine ring proton); 3.16 (q, 4H, J = 7.2 Hz, N-C**H**_2_-CH_3_), 3.10 (d, 2H, J = 13.2 Hz, piperazine ring proton); 3.07 (d, 2H, J = 12.8 Hz, piperazine ring proton); 2.825 (s, 3H, N-CH_3_); 2.81 (d, 2.2 H, J = 19.2 Hz, citrate); 2.67 (d, 2.2H, J = 15.6 Hz, citrate); 1.02 (t, 6H, J = 7.2 Hz, N-CH_2_-C**H**_3_). The citrate methylene peaks overlapped the N-CH_3_ group on the DEC, so this spectrum was not used to calculate the citrate:DEC stoichiometry. DEC citrate in DMSO (∼16 mg/ml, 400 MHz, shifts in ppm vs. TMS): ∼10.5 (br s, 3.17H, R_3_N**H** and COO**H** groups); 3.17(m, 4H, J = 4.8 Hz, piperazine ring proton); 3.11 (q, 4H, J = 7.2 Hz, N-C**H**_2_-CH_3_), 2.75(m, 4H, J = 4.8 Hz, piperazine ring proton); 2.63 (d, 2.05 H, J = 15.6 Hz, citrate); 2.59 (d, 2.05 H, J = 15.2 Hz, citrate); 2.49 (s, 3H, N-CH_3_, this peak is superposed directly on the DMSO residual, which is clearly not a normal -CD_2_H pentet peak); 1.03 (t, 6H, J = 7.2 Hz, N-CH_2_-C**H**_3_).

#### Analysis of DEC citrate by back titration

A 10.00 ml aliquot of the solution to be analyzed is mixed with 10.00 ml of 0.0100 M NaOH and two drops of phenolphthalein indicator are added to give a uniform pink color. The solution is titrated to a clear endpoint with 0.00400 M HCl. The titration should be carried out in triplicate and the results averaged; the relative standard deviation of the endpoint volumes for a triplicate trial 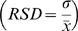 should be 0.02 or less (typically 0.005).

#### Analysis of DEC citrate by HPLC

Samples were analyzed using Mathew's method [12] on a Shimadzu HPLC. The column was a 15 cm×4.6 mm Luna C8 column (Phenomenex) of 5 µm particle size with a pre-column fritted filter and a 0.50 ml/min flow rate; column pressure was about 1200–1400 psi during the run. The eluent was 9 parts of phosphate buffer (20 mM KH_2_PO_4_ adjusted to pH 3.2 with H_2_SO_4_ or H_3_PO_4_) and 1 part acetonitrile. The eluent was degassed by vacuum filtration through a 0.4 micron ceramic filter. The detector wavelength was set at 210 nm and a 20 µl sample loop was used. DEC citrate elutes at approximately 5.6 minutes.

Samples and standards prepared for back titration are approximately 10% salinity, with 100 mg/ml NaCl, which would clog the HPLC column. These samples were diluted 10X and filtered on a Whatman GD/X 0.45 µm PES syringe filter before injection onto the HPLC column. Using this sample preparation protocol, a standard salt sample containing 0.25% w/w DEC, when prepared for analysis on the HPLC, contains 0.025 mg/ml DEC citrate and 10 mg/ml NaCl. Standards for HPLC analysis ranged from 0.005–0.200 mg/ml DEC citrate in 1.0% saline.

## Results/Discussion

### Chemical basis of the analysis

**Figure 1:**
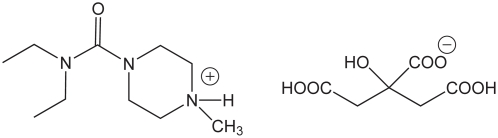
DEC citrate.

Standard DEC citrate used in this study (from Sigma-Aldrich) was identical by NMR (spectra acquired in D_2_O and d_6_-DMSO at 400 MHz) to a sample of the DEC citrate (manufactured by EPICO) that is used at the Bon Sel factory in Haiti. The 1∶1 DEC∶citrate stoichiometry was confirmed by integration of the ^1^H-NMR peaks from the diastereotopic methylene groups on the citrate and the triplet from the ethyl groups on the DEC (predicted for a one-to-one stoichiometry of DEC∶citrate: 4∶6, found 4.2∶6.0.) From the DEC∶citrate stoichiometry, each equivalent of DEC citrate (see structure in Figure 1) contains three acidic protons (two carboxylic acids and one protonated tertiary amine). These three acidic protons are visible as a very broad peak at 10.5 ppm when the spectrum is acquired in dry DMSO-d_6_.

Direct titration of DEC citrate with base did not prove analytically useful. Due to the range of pKa values in the polyprotic citrate, the end point of the titration was not clear enough. However, back titration gave a clear endpoint. In the back titration, a sample of DEC citrate is added to a known excess of the strong base sodium hydroxide, which reacts completely with the acidic protons. The remaining hydroxide is titrated with standard HCl, giving a clear endpoint with the common indicator phenolphthalein. Bon Sel also contains small amounts of potassium iodate to supply 40 ppm iodine as a nutritional supplement. Calibration with DEC citrate standards compensates for any matrix effects from the salt or interference from the iodate. It should be noted that this analytical method is not as specific or generally useful as the HPLC analysis, because any acidic or basic compound will interfere with the back-titration. Thus, this test cannot be applied to complex matrices (e.g., determination of DEC concentration in cooked food or in body fluids).

### Titration results

**Figure 2:**
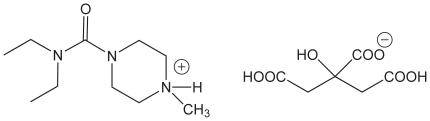
DEC citrate standard curve for back titration. DEC concentration (g/100 g salt) versus endpoint volume of back-titration are plotted for triplicate standards.

Titration of standard samples gave a linear calibration curve (Figure 2); the linear least-squares parameters were determined in Excel using the LINEST function and used to fit unknown samples. The linear range extends from 0.050% to 0.88% (w/w DEC citrate in salt), which covers the normal therapeutic range of DEC in salt (0.1–0.6%, recommended 0.2–0.4%). [17] The average relative standard deviation (RSD) for the concentration of known DEC samples at Notre Dame was 16±9% by the titration method, based on triplicate analysis of samples ranging from 0.10% to 0.90% DEC citrate. Samples analyzed in Haiti gave an average RSD of 33±7%. The limit of detection (LOD = 3*s/m) and limit of quantification (LOQ = 10*s/m) were calculated; [18] *m* is given by the least square fit to the slope of the calibration curve, and *s* is the standard deviation of 7 determinations of DEC concentration for the 0.050% standard sample. The LOD is 0.029% and the LOQ is 0.096% for the titration method.

**Figure 3:**
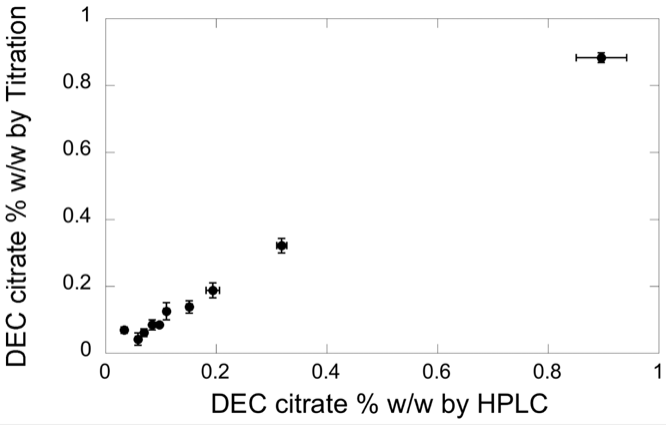
Comparison of back-titration and HPLC concentration determinations. Error bars show standard deviation of triplicate measurements. For low concentration samples, error bars in HPLC measurement are very small.

To compare the titration method and the HPLC method, multiple standards and unknowns were analyzed with both methods. Figure 3 shows the results plotted against each other; the observed slope of the line is 1.014 (for perfect agreement it would be 1.00). The accuracy of the titration method was indistinguishable from that of the HPLC method. Applying the paired t-test [19] for the 10 samples listed in Table 1, the mean difference between the titration and HPLC results was −0.0018, the std deviation was 0.016, and t_calc_ is 0.35. This indicates that the difference between the titration and HPLC results was not statistically significant for samples at concentrations of 0.1%–0.8%, although the precision of the HPLC method was superior (RSD <5% for HPLC) and its LOQ was much lower.

**Table 1:** Comparison of titration and HPLC analysis of Bon Sel samples.

Titration average	HPLC average	Difference
(%DEC citrate w/w)	(%DEC citrate w/w)	
0.0419±0.0183	0.0591±0.0047	−0.017
0.0614±0.0114	0.0696±0.0011	−0.008
0.0692±0.0096	0.0342±0.0010	0.035
0.0848±0.0069	0.0970±0.0020	−0.012
0.0848±0.0150	0.0846±0.0022	0.0002
0.1253±0.0254	0.1105±0.0005	0.015
0.1381±0.0183	0.1516±0.0049	−0.014
0.1881±0.0220	0.1941±0.0122	−0.006
0.3211±0.0215	0.3185±0.0092	0.0026
0.8835±0.0145	0.8965±0.0456	−0.013

Analysis of Bon Sel samples from seven production runs in mid-2009 showed that all seven production lots ranged from 0.09–0.13% DEC citrate, with an average of 0.10%±0.01%. (Table 2) This shows that spray coating is an effective technique for achieving uniform DEC loading on salt at the kg-to-kg and lot-to-lot level. The loading achieved, while in the therapeutic range (0.1–0.6% w/w), was lower than the desired loading of 0.2–0.4% w/w. The loading is a function of the solubility of the DEC citrate in the spraying solution, the drying rate of the salt, and the salt feed rate, and could not be improved with the equipment on hand. However, the group in Haiti tried an experimental run where a finished batch of salt was dried and fed back into the sprayer; this double-sprayed salt analyzed at 50±7 ppm iodine and 0.28±0.7% w/w DEC citrate (Table 2, entries X1 (single sprayed) and X2 (double sprayed)).

**Table 2:** Within-lot and between-lot DEC citrate concentrations* in Bon Sel samples determined by titration method.

Lot #	Sample 1	Sample 2	Sample 3	Lot Average
	% Concentration DEC	% Concentration DEC	% Concentration DEC	
**1**	0.130±0.046 (Haiti)			
**11**	0.177±0.051 (Haiti)			
**16**	0.085±0.007	0.081±0.016	0.110±0.016	0.092
**17**	0.131±0.007	0.08±0.01	0.09±0.02	0.098
**18**	0.09±0.03	0.12±0.02	0.10±0.01	0.103
**19**	0.133±0.009	0.106±0.015	0.084±0.025	0.108
**20**	0.12±0.01	0.09±0.02	0.187±0.015	0.133
**21**	0.09±0.01	0.1083±0.0016	0.132±0.035	0.110
**22**	0.072±0.006	0.11±0.03	0.09±0.03	0.092
**X1**	0.05±0.02 (Haiti)			
**X2**	0.28±0.07 (Haiti)			

*Different samples were taken from different bags of Bon Sel (see text for discussion of sample heterogeneity). The lots are approximately 500 kg in weight. Errors for each sample are the standard deviations for triplicate titration of the sample, except for X1, which was titrated 6 times. Lots 16–22 were analyzed by the titration method at Notre Dame, the other samples were analyzed in Haiti.

To monitor heterogeneity within the bags of Bon Sel, three 10 g grab samples from each of several 1 kg bags of Bon Sel (taken from different lots) were tested; the levels of DEC citrate varied from 0.08 to 0.15% for samples taken within the same bag of Bon Sel. This heterogeneity was not due to errors in the titration analysis, as the results were confirmed by HPLC analysis, which has a much higher precision. Because the DEC is sprayed onto the salt, which contains both coarse (low surface area) and fine (high surface area) crystals, DEC loading is expected to be a function of salt crystal size. Two lots of Bon Sel from the mid-2009 production runs were screened to separate particles >4 mm in size from particles <4 mm in size; in each case, the large crystals had significantly lower loading of DEC than the small crystals. For example, in one lot, the large crystals gave a DEC loading of 0.034±0.001% while the small crystals came in at 0.085±0.002% (these low loadings were measured using HPLC to obtain more precise results). The variation in loading with crystal size appears to be large enough to account for most of the heterogeneity in the within-lot analyses, and suggests that more uniform spray coating and higher loadings would be achieved by crushing the salt before spraying it.

The salt available in Haitian markets is often of low purity, and many people rinse the salt before using it in cooking. Although Bon Sel is pre-washed and the packaging advises consumers not to wash the salt, habits can be hard to break, and some people probably still wash the Bon Sel. Tests on the effect of hand rinsing (∼5 seconds swirling in a bowl of water, or a similar time under a stream of water) showed retention of 40–50% of the DEC citrate and 60–70% of the iodate after the medicated salt was washed. This result suggests that a fortification level of 0.3–0.4% DEC citrate, at the high end of the recommended scale, would be likely to deliver therapeutically useful doses to consumers of the medicated salt regardless of whether or not they rinse it.

### Conclusions

A simple titration-based assay allows determination of diethylcarbamazine (DEC) citrate concentrations in medicated salt produced in Haiti for an anti-lymphatic filariasis program. The assay can be carried out with widely available equipment and materials and thus offers a useful tool for quality control and field analysis of DEC. The development of this method, which allows quantification of the medication, DEC citrate, has already proven useful for quality control in the Haiti plant where salt fortification takes place. Historically, identification and communication of flaws in the salt fortification levels have taken several months as samples were sent back to the US for analysis. Using the back titration analysis of DEC, chemists in Haiti can now identify variation in DEC loading as batches of Bon Sel are produced. This analysis will allow the Bon Sel plant to act more rapidly and independently in their effort to supply the area with properly medicated salt. An increased efficiency in Bon Sel production should bolster the endeavor to reduce and eventually eliminate lymphatic filariasis in Haiti.
